# Divergence in leaf and cambium phenologies among three temperate tree species of different wood types with special reference to xylem hydraulics

**DOI:** 10.3389/fpls.2025.1562873

**Published:** 2025-03-03

**Authors:** Ai-Ying Wang, Si-Qi Li, Han-Xiao Cui, Ya-Nan Liu, Yi-Jun Lu, Guang-You Hao

**Affiliations:** ^1^ College of Life Science and Bioengineering, Shenyang University, Shenyang, Liaoning, China; ^2^ Liaoning Key Laboratory of Urban Integrated Pest Management and Ecological Security, College of Life Science and Bioengineering, Shenyang University, Shenyang, Liaoning, China; ^3^ CAS Key Laboratory of Forest Ecology and Silviculture, Institute of Applied Ecology, Chinese Academy of Sciences, Shenyang, China; ^4^ College of Resources and Environment, University of Chinese Academy of Sciences, Beijing, China

**Keywords:** cambial phenology, leaf phenology, winter embolism, xylem formation, xylogenesis

## Abstract

Leaf and cambium phenologies are both important aspects of tree environmental adaptation in temperate areas. Temperate tree species with non-porous, diffuse-porous and ring-porous woods diverge substantially in the strategy of coping with freezing-induced hydraulic dysfunction, which can be closely associated with the timing of both leaf phenology and xylogenesis. Nevertheless, we still know little about the potential differences in the intra-annual process of xylogenesis among species of the three functional groups as well as its association with leaf phenology. Here, we monitored leaf phenology and xylogenesis in a non-porous (*Pinus*), a diffuse-porous (*Populus*), and a ring-porous (*Ulmus*) temperate tree species in a common garden. The results showed clear divergences in leaf and cambium phenologies and their chronological orders among the three species. The two hardwood species exhibited earlier bud burst and leaf unfolding than the conifer. The cambial activity of the ring-porous species began earlier than the diffuse-porous species, although the leaf phenology of the diffuse-porous species was earlier. The conifer species showed the latest bud break but the initiation of cambium activity was the earliest, which can be attributed to its strong resistance to freezing-induced embolism in the tracheid-based xylem. The leaf phenology preceded the onset of cambial activity in the *Populus* species, which was permitted by the ability of diffuse-porous species in largely retaining the stem hydraulic function over the winter. In contrast, the *Ulmus* species with ring-porous wood had to restore its severely hampered stem hydraulic function by winter embolism before leaf flush. The results revealed that leaf and cambium phenologies are closely interconnected due to the coordination between xylem water transport and leaf water demand. These findings contribute to a better understanding of the divergent adaptive strategies of temperate trees with different wood types.

## 1 Introduction

Plant phenology refers to the timing of annually recurring plant life cycle stages in response to seasonal climatic changes, including the timing of bud burst, leaf unfolding, flowering, fruiting, and cambial activity, etc., and is a critical aspect of plant adaptation to their environments (Linkosalo et al., 2009; Morel et al., 2015). It reflects the balance that plants strive to achieve between optimizing growth potential and minimizing risks from adverse climate conditions (Delpierre et al., 2016). In seasonally cold environments, temperate deciduous trees avoid cold stress by regulating the timing of spring bud burst, as well as leaf senescence and abscission in autumn (Badeck et al., 2004). This periodic rhythmic change reflects a strategy for trees to maximize resource utilization and minimize risks related to low temperatures (Chuine, 2010; Nord and Lynch, 2009). Due to seasonal changes of climate conditions, the cambium also alternates between active and dormant periods over the year (Qian et al., 2023; Rossi et al., 2016, 2006b). During spring and summer, the cambium is active, taking advantage of warm and humid conditions for stem growth. In autumn and winter, cambial activity decreases or ceases, and the metabolic processes of trees slow down as temperature drops (Begum et al., 2016). Leaf phenology and cambium phenology are likely coupled but research linking the two remains relatively scarce (Lavrič et al., 2017; Savage and Chuine, 2021). One of the main functional association between cambium and leaf phenologies can be related to the fact that the growth of stem xylem in spring is vital for trees to rebuild an effective water transport system required for leaf transpiration, which may have been severely damaged due to the freezing-induced embolism over the winter (Hao et al., 2013; Niu et al., 2017; Sperry and Sullivan, 1992; Yin et al., 2018). It remains largely unclear what the mechanisms linking leaf and cambium phenologies under temperate climate conditions are, and how the coupling of leaf and cambium phenologies influence species’ adaptive strategies with respect to the coordination of plant water relations and carbon economics, although this becomes particularly important as global warming are significantly influencing various aspects of plant phenology (Du et al., 2018; Morin et al., 2010; Muffler et al., 2024).

The leaf and cambium phenologies are regulated by internal factors, such as hormones, and are functionally closely coupled as leaf and stem xylem are two key components of water flux system through plants. The onset of spring cambial activity is controlled by auxin produced by buds and growing leaves, although the sensitivity of cambial reactivation to auxin can be different among species (Aloni, 1992, 2021). In diffuse-porous tree species, cambial activity requires a high concentration of auxin, from rapidly growing young leaves, to be reactivated and to extend from the branches down to the base of the trunk (Takahashi et al., 2013). In contrast, ring-porous tree species experience extremely rapid cambial reactivation before bud burst with this process occurring almost simultaneously in both the trunk and branches, which is attributable to their sensitivity to low levels of auxin originating in dormant-looking buds a few weeks before bud break (Aloni, 1991; Aloni and Peterson, 1997; Aloni et al., 1997; Puchałka et al., 2017; Takahashi et al., 2013). In conifer species, bud burst may occur either before, after or synchronized with the production of stem xylem cells (Antonucci et al., 2015; Cuny et al., 2012; O’Reilly and Owens, 1989). However, the adaptive significance of the relationship between leaf and cambium phenologies remains uncertain across different tree functional groups, particularly with respect to the coordination between xylem water transport and leaf transpirational water demand. Leaf flush in spring leads to an abrupt increase in tree water demand, which necessitates that the xylem at least maintains some functionality when bud burst begins (Lavrič et al., 2017). Coniferous and diffuse-porous species are less susceptible to freezing-induced xylem embolism and still maintain a largely functional hydraulic system over the winter (Davis et al., 1999), while ring-porous species adopt a strategy of “abandoning” the heavily winter-emblized vessels and produce new ones in spring to meet the water transport demand (Hacke and Sauter, 1996; Sperry et al., 1994). Therefore, from the perspective of the functional coordination between xylogenesis and hydraulics, studying the chronological sequences of leaf and cambium phenologies can help us better understand the adaptive strategies of different temperate tree species, particularly among species of different wood types.

The xylogenesis involves the division and differentiation of cambial cells, such as cell enlargement, secondary wall thickening, and the maturation of xylem cells (Perrin et al., 2017; Qian et al., 2023; Rossi et al., 2006b, 2012). This process is regulated not only by hormones but also by the water and carbon status of plants. It has been shown that water status controls xylem cell production, while carbon is the energy source of cambial activity and cell differentiation (Deslauriers et al., 2016; Michelot et al., 2012). Changes in the coupled water and carbon relations of trees can likely affect the process of xylem formation (Jyske and Holtta, 2015; Steppe et al., 2015). The hydraulic architecture of trees influences their ability to transport water from the soil to the leaves, which in turn affects photosynthetic carbon assimilation and growth rate (Tyree and Ewers, 1991). Numerous studies have explored the relationship between xylem hydraulics and tree growth from the perspective of water-carbon coupling, showing that species with higher hydraulic efficiency tend to have faster growth rates (Fan et al., 2012; Wang et al., 2022). Additionally, in temperate regions, the divergence in strategies of coping with the risk of freezing-induced xylem embolism may lead to differentiation in radial growth patterns. The tracheid-based xylems usually exhibit strong resistance to embolism induced by freeze-thaw cycles due to their small conduit sizes but may have limited the potential of water transport efficiency in conifers, although the low end wall resistance to water flow due to the torus-margo pit structure allow some of them have comparable hydraulic efficiency to angiosperm trees (Davis et al., 1999; Pittermann et al., 2005; Sperry et al., 1994). Thus, conifer trees may have slow instantaneous growth rates but have longer growth periods during a year. In contrast, deciduous angiosperm tree species have larger and wider vessels that improved water transport efficiency but are at the cost of reduced resistance to freezing-induced embolism (Yin et al., 2022). These species may adopt a quick growth strategy during their shorter growth period. In particular, for ring-porous species, although their leaf phenology is usually later than that of diffuse-porous species, their larger early-wood vessels enable them to have higher water transport efficiency (Song et al., 2022; Wang et al., 2022; Yang et al., 2019). This might allow them to utilize their higher water transport efficiency and higher photosynthetic carbon assimilation capacity after bud burst, achieving higher growth increment in a shorter growth period. However, few studies have compared intra-annual radial growth patterns among coniferous, diffuse-porous, and ring-porous tree species with different xylem structures, and even fewer have discussed their differences from the perspective of hydraulic adaptation.

In this study, using field observations and microcore analyses we monitored the leaf and cambium phenologies in the trunks and branches of *Pinus tabuliformis* Carrière, *Populus alba* L. × *P. berolinensis* Dippel, and *Ulmus pumila* L., i.e. three important temperate tree species commonly found in northern China. Particularly, the three species represent trees of different wood types, including non-porous (*P. tabuliformis*), diffuse-porous (*P. alba* × *P. berolinensis*), and ring-porous (*U. pumila*) tree species. Such difference makes them highly representative for comparing environmental adaptation strategies of distinct tree functional groups. This study focused on the chronological sequence between leaf and cambium phenologies in trees with different wood types, as well as the role of their coordination in shaping the water-carbon coupling strategies of temperate trees. We proposed the following specific hypotheses: (I) The deciduous species bud burst earlier than the evergreen species, and the diffuse-porous species bud burst earlier than the ring-porous species. (II) The difference in chronological order of leaf and cambium phenologies among tree species of different wood types stems from the coordination between stem hydraulics and leaf transpirational water demand along with seasonal changes. (III) The non-porous species would have a longer xylem growth period and slower growth rate, while the diffuse-porous and the ring-porous species exhibit a more concentrated xylem growth over a shorter period.

## 2 Materials and methods

### 2.1 Study site and plant materials

The study was performed in Qingnian Park (41.78°N, 123.44°E), located in Shenyang, Liaoning Province, Northeast China. The region has a temperate sub-humid monsoon continental climate. Daily climate data, including temperature, precipitation and relative humidity in 2023, were obtained from Shenyang Arboretum, Chinese Academy of Sciences, which is about 2 km from the study site. Vapor pressure deficit (VPD) was calculated based on relative humidity and temperature data using the RHtoVPD function in R package ‘plantecophys’ (Duursma, 2015). In 2023, the mean temperature was 9.71°C, with the highest and lowest temperature of 32.78°C (25th June) and -23.87°C (23rd December), respectively. Total precipitation in 2023 was 528.9 mm, with approximately 83% occurred from April to September. Relative humidity ranged from 19.5% to 97.30% (**Supplementary Figure S1**).

Three upright and healthy individuals of each of the three tree species were chosen to monitor leaf and cambium phenologies. Trees of the three tree species were growing under similar environmental conditions of the common garden. The average diameters at breast (DBH) were 24.2 ± 3.2 cm, 23.3 ± 1.1 cm, and 37.9 ± 5.3 cm for *Pt*, *Pab*, and *Up*, respectively and the tree ages ranged from 15 to 25 years.

### 2.2 Leaf phenological observations

Leaf phenology was observed on the three tree species at 2-day intervals from March to November 2023. Following the method of Gričar et al. (2017), we observed the leaf phenology of the two deciduous broadleaved tree species according to a seven-phases scale: 1-swollen bud, 2-bud break, 3-leaf emergence, 4-leaf development, 5-full leaf unfolding, 6-initiation of autumn colouring (the beginning of leaf colour change from green to yellow, red and orange), and 7-leaf fall. The spring phenology of the conifer species was described as five phases: 1-swollen bud (smooth and pale scales but invisible needles), 2-translucent bud (visible needles through the scales), 3-split bud (open scales and cluster needles), 4-exposed shoot (fully emerging needles), and 5-leaf fully expanded (fresh needles reaching half the length of old needles), according to a protocol adjusted from Perrin et al. (2017). The autumn leaf phenology of the evergreen conifer species was not observed due to a lack of obvious changes in the number and colour of leaves.

### 2.3 Cambium phenology and xylogenesis monitoring in tree trunk and branches

In 2023, microcores of branches and trunks were collected weekly from March to June, biweekly from July to August, and monthly from September to October using a Trephor tool (Rossi et al., 2006a). For branches, the microcore samples were taken in the stems 1-2 m from the branch apex (Gričar et al., 2017). The sampled branches, with diameters at the sampling locations of 2-3 cm, were located approximately 3 m above the ground. A long-reach pruning shear was used to obtain a branch from each tree, and then Trephor was used to obtain microcores on the branches. All branches were sampled from the southern side of selected trees. To minimize the effect of high-frequency sampling of branches on tree growth, we sampled 7-8 individuals for collecting branch microcores per species in addition to the trees that were used for leaf and trunk phenological monitoring. The sampling trajectory from trunk at breast height (1.3 m) followed a spiral pattern, with an approximate 3 cm separation between each sampling to minimize the wound effect. Wood microcores were preserved in Formalin-Aceto-Alcohol (FAA) solution and stored at 4°C to avoid tissue deterioration. After 1-2 weeks, the samples were dehydrated in a graded series of ethanol (70%, 90%, 95%, 100%), infiltrated with D-limonene and embedded in paraffin blocks. Transverse sections of 12-16 μm were cut with a rotary microtome (YD-335 A, Jinhua YIDI Medical Appliance Co., Ltd., Zhejiang Province, China). These sections were cleared in D-limonene and 100% ethanol, stained in a safranin water solution (0.04%) and astra blue water solution (0.15%). Cambium cells and xylem cells can be clearly distinguished by staining. Usually, lignified cells (mature xylem cells) are stained red or purple, and non-lignified cells (cambium cells) are stained blue. Then the samples were photographed under an optical microscope (DM750, Leica, Wetzlar, Germany).

**Figure 1:**
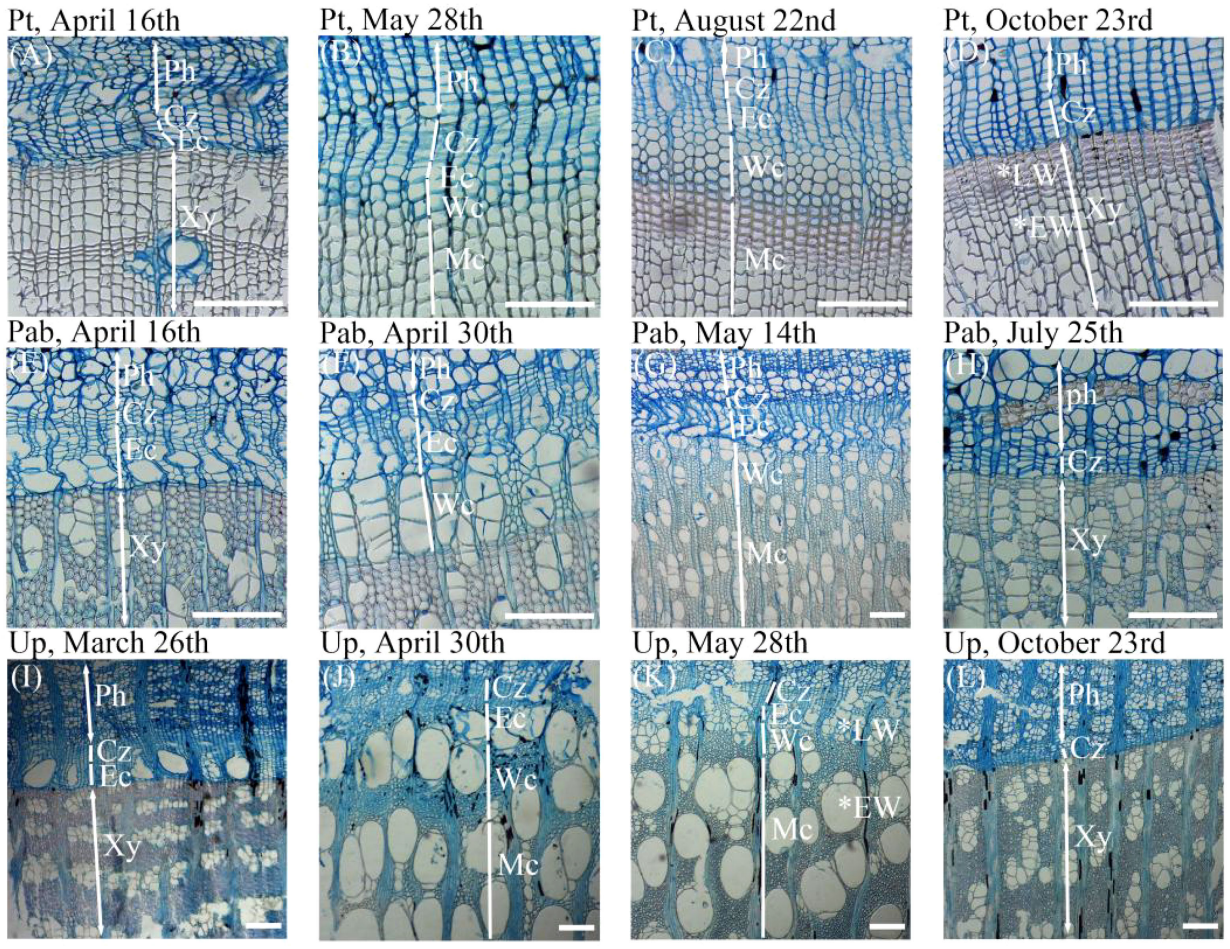
Types of cells corresponding to different phases of xylem formation observed in the growing season of 2023 for the three studied tree species. **(A–D)** The xylem cell morphology of Pinus tabuliformis in April, May, August, and October. **(E–H)**The xylem cell morphology of Populus alba ×P. berolinensis in April, May, and July. **(I–L)** The xylem cell morphology of Ulmus pumila in March, April, May, and October. Ph, phloem; Xy, Xylem; EW, early wood; LW, late wood; Cz, cambium cell zone; Ec, enlarging cell; Wc, wall-thickening cell; Mc, mature cell. The white horizontal bars = 200 μm.

Based on the cell morphology and the staining characteristics of the cell wall (**Figure 1**), we measured the widths of cambial cell zone, enlarging cell zone, wall-thickening cell zone and maturing cell zone along three radial files in each sample using ImageJ software (https://imagej.nih.gov/ij/). Cambial cells and enlarging cells were both stained blue, but cambial cells had thinner walls and smaller radial diameters. During cell enlargement, the radial diameters of enlarging cells were approximately twice those of cambial cells (Zhang et al., 2018). Wall-thickening cells showed red outer walls and blue inner walls indicating incomplete maturity. Mature cells, with lignified walls and lacking protoplasts were completely stained in red. In our study, cambium phenology was assessed as the dates of first enlarging cell, first wall-thickening cell, first mature cell, last enlarging cell and last wall-thickening cell (Perrin et al., 2017).

### 2.4 Data analysis

The dates of leaf, branch and trunk phenology were expressed as day of year (DOY). Analysis of variance (ANOVA) and Tukey’s tests were used to test the differences among the three species for leaf, branch and trunk phenology in SPSS 22.0. The differences in branch and trunk phenology within a species were compared using the *t*-test. For each species, the mean cumulative radial growth and the radial growth rate were fitted by the Gompertz function (Equation 1) using the following formula (Michelot et al., 2012):


(1) y=A exp[-e(β-κt)]

where y is the cumulative xylem width (µm), t is the day of year, A is the upper asymptote (µm), β is the placement parameter, and κ is the rate of change parameter. All figures were completed in Origin Lab 2018.

## 3 Results

### 3.1 Inter-specific differences in leaf phenology

**Figure 2:**
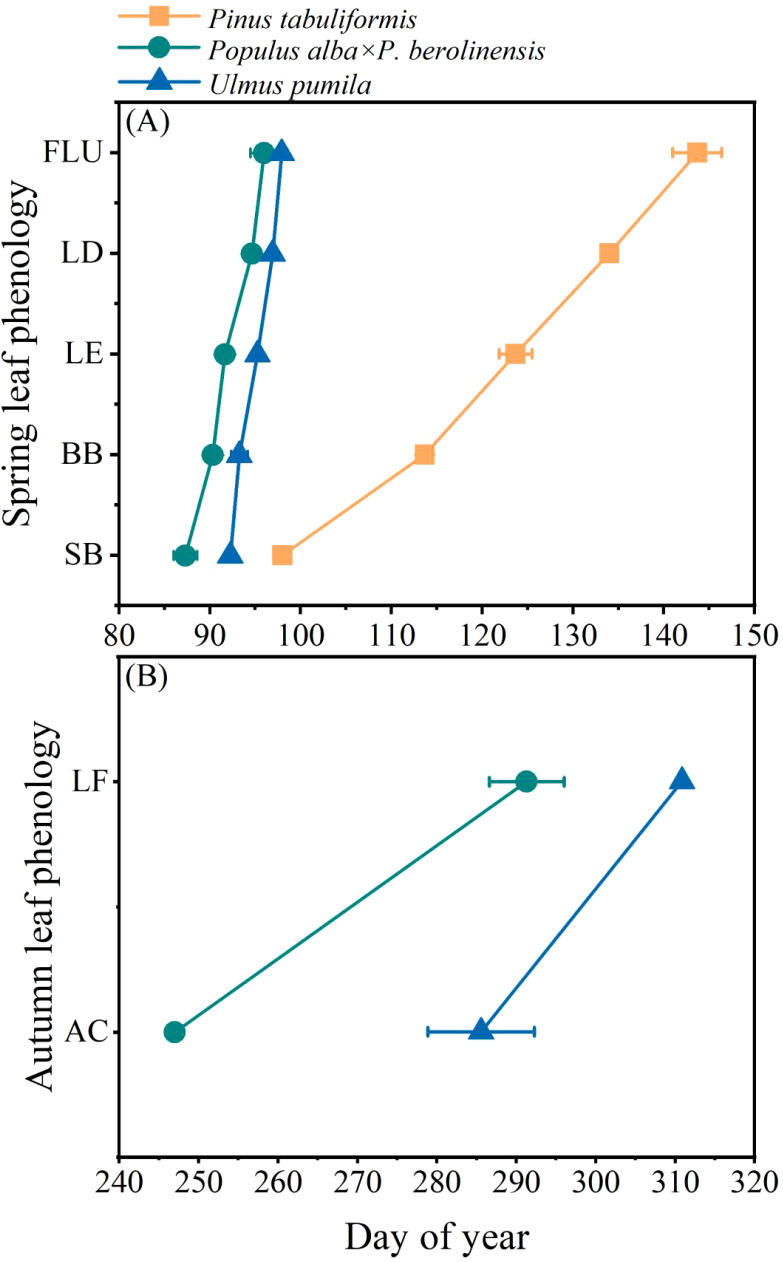
The occurrence time of different phases of leaf phenology of the three studied tree species. **(A)** Spring leaf phenology and **(B)** Autumn leaf phenology. SB, swollen buds; BB, bud break; LE, leaf emergence; LD, leaf development; FLU, full leaf unfolding; AC, initiation of autumn colouring; LF, leaf fall. Data are presented as mean ± 1 SE.

There were distinct differences in leaf phenology among the three tree species with different wood types (**Figure 2**, **Supplementary Table S1**). Overall, the two broadleaved tree species had much earlier leaf phenology than the conifer species. During the early spring of 2023, bud expansion occurred in the *Populus* species in late March (DOY 87.3), followed by *U. pumila* that was approximately one week later (DOY 92.3). Both species exhibited rapid leaf unfolding and only took 4-5 days from bud break to full leaf unfolding. In contrast, *P. tabuliformis* showed a delayed bud expansion (DOY 98.0), and its leaf growth extended until late May (DOY 143.7). For the autumn leaf phenology of the two deciduous species, the *Populus* species*-initiated* leaf coloring on DOY 247.0 and the complete leaf abscission occurred about a month later (DOY 285.7). *U. pumila*, showed a much later onset of leaf coloring (DOY 291.0) and complete leaf fall occurred until early November (DOY 311.0).

### 3.2 Inter-specific differences in cambium phenology of the trunks

**Figure 3:**
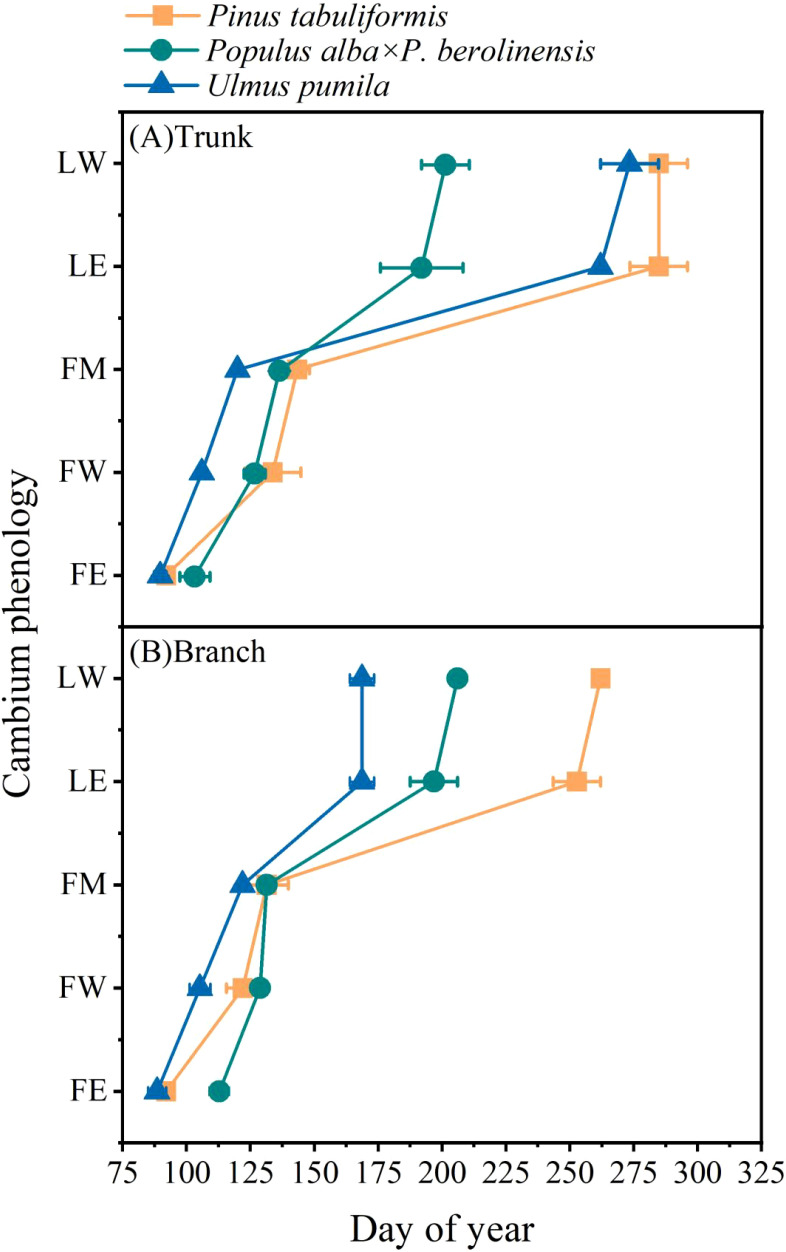
The occurrence time of different phases of cambium phenology of the three studied tree species. **(A)** Cambium phenology in the trunk and **(B)** Cambium phenology in branches. FE, first enlarging cell; FW, first wall-thickening cell; FM, first mature cell; LE, last enlarging cell; LW, last wall-thickening cell. Data are presented as mean ± 1 SE.

**Figure 4:**
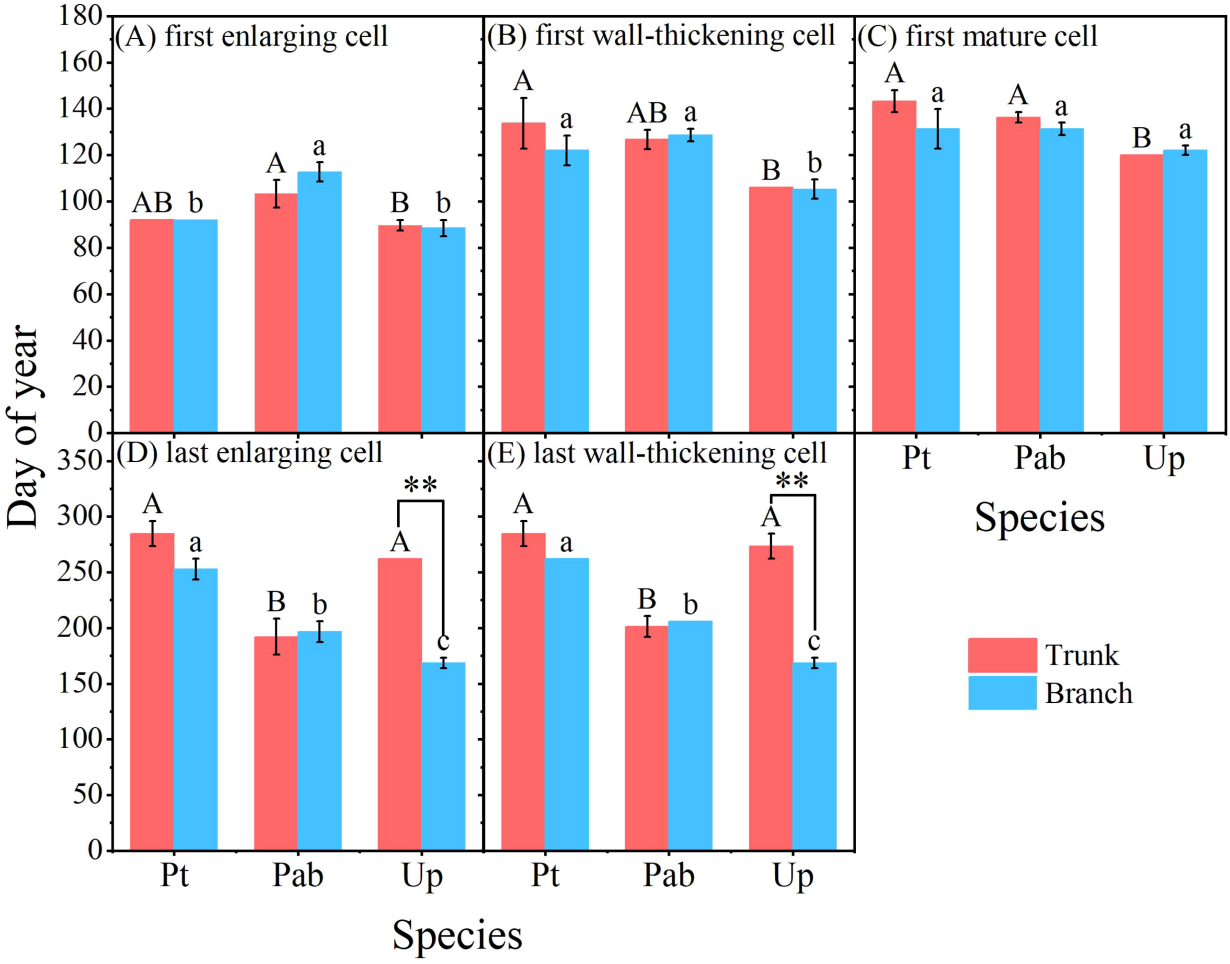
Cambium phenology in the trunk and branches of the three studied tree species in 2023. **(A–E)** The timing of each phase of the cambial phenology occurring in the trunks and branches (including first enlarging cell, first wall-thickening cell, first mature cell, last enlarging cell, and last wall-thickening cell). Different upper and lower case letters on top of the bars indicate significant differences (*P* < 0.05) among the three species in trunk and branches, respectively. The “**” on top of the bars indicate significant difference between trunk and branch within a species (*P* < 0.001). Data are presented as mean ± 1 SE.

Clear differences in the timing of cambium phenology of the tree trunk were also identified among the three tree species (**Figures 3A**, **4A–E**). The first enlarging cell occurred in the tree trunk of *U. pumila* began the earliest (DOY 89.7) with *P. tabuliformis* closely followed (DOY 92.0), while *P. alba* × *P. berolinensis* had the latest onset of trunk cambium activity (DOY 103.3). In addition to the timing of the appearance of the first wall-thickening cell, there were significant differences in the timing of each phase of xylogenesis between the diffuse-porous and the ring-porous species (**Figures 4A–E**, **Supplementary Table S2**). The duration of xylogenesis in *P. tabuliformis* and *U. pumila* lasted over three months, nearly double the duration observed in *P. alba × P. berolinensis* (**Figure 3A**).

### 3.3 Difference in cambium phenology between trunks and branches

In *P. tabuliformis*, cambial activity in the trunks and branches began at the same time (DOY 92.0), but the trunk’s cambial activity ended 3 weeks later than that in the branches, i.e. DOY 284.7 vs. DOY 262.0, respectively. In *P. alba* × *P. berolinensis*, cambial activity in the trunks (DOY 103.3) began 1 week earlier than that in the branches (DOY 112.7), and ended 5 days earlier (DOY 201.0) than that in the branches (DOY 206.0), resulting in a nearly equal duration of xylogenesis in the trunks and branches. In *U. pumila*, the cambial activity in the trunks (DOY 89.7) and branches (DOY 88.5) began almost simultaneously (**Figures 3A, B**). However, a significant difference was observed in the timing of the cambial activity cessation (**Figures 4D, E**), with the branches (DOY 168.7) being much earlier than the trunks (DOY 273.7).

### 3.4 Comparison between leaf phenology and stem cambium phenology

**Figure 5:**
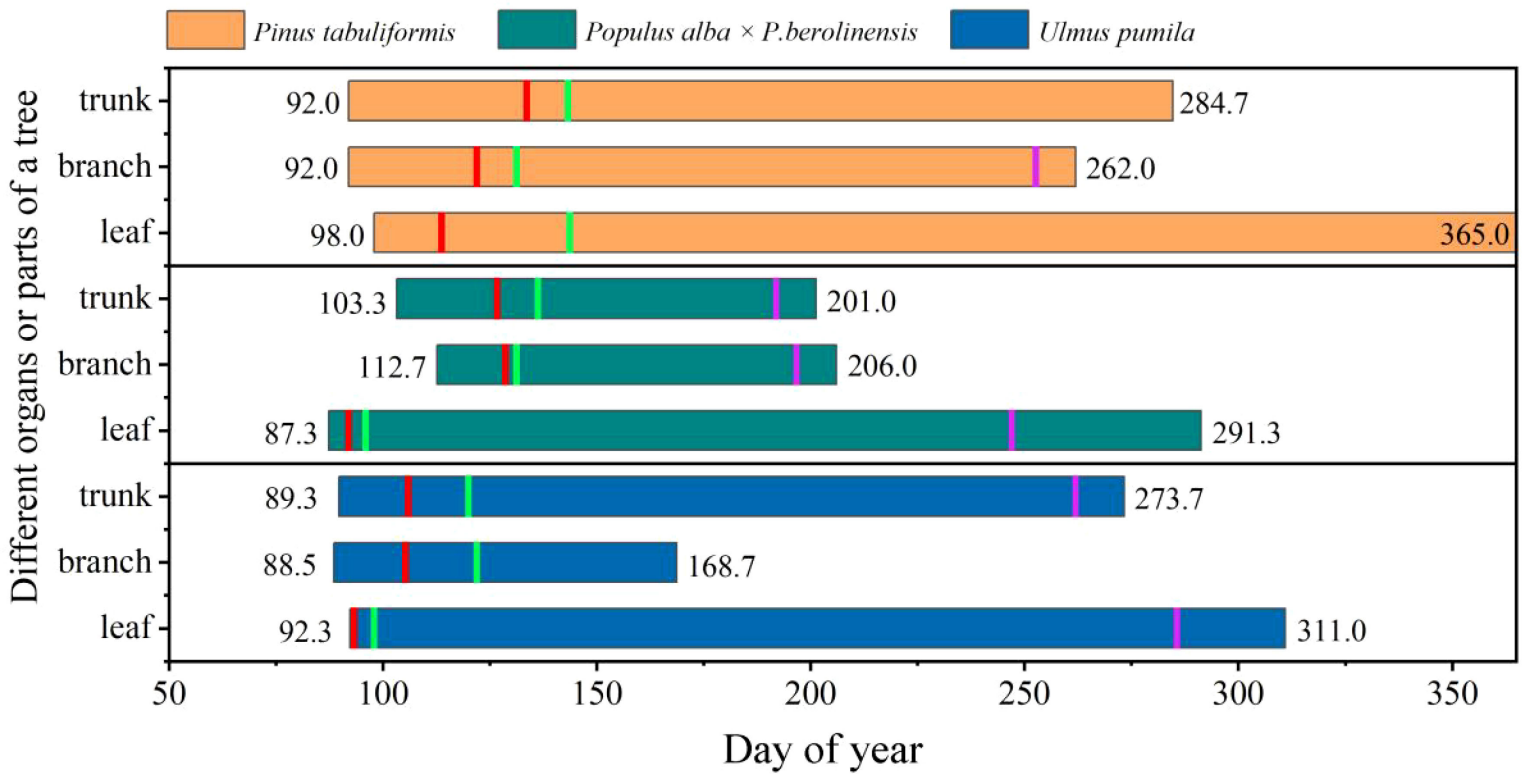
Leaf and wood growing periods of the three studied species in 2023, defined by corresponding phenological terms. The short vertical lines represent the time of each phase of leaf and cambium phenology. For the trunk and branches, the vertical lines from left to right represent the time of the first enlarging cell (left boarder of the rectangle), the first wall-thickening cell (red), the first mature cell (green), the last enlarging cell (purple) and the last wall-thickening cell (right boarder of the rectangle). For the leaves, the vertical lines represent swollen buds (left boarder of the rectangle), bud break (red), full leaf unfolding (green), initiation of autumn colouring (purple), and leaf fall or death (right boarder of the rectangle), respectively.

There were clear differences in the chronological sequence between leaf phenology and cambium phenology in the trunks and branches among the three species (**Figure 5**). In *P. tabuliformis*, the initiation of cambial activity (first enlarging cell) in both the trunks and branches occurred simultaneously (DOY 92.0) and was 21 days prior to its bud burst (DOY 113.7). In *P. alba* × *P. berolinensis*, bud burst occurred on DOY 92.0, which was 10 and 20 days before the initiation of cambial activity (first enlarging cell) in the trunks (DOY 103.3) and the branches (DOY 112.7), respectively (**Figure 5**, **Supplementary Table S2**). The xylem lignification in this species did not begin until the leaves were fully unfolded. About 1 month after the cambial activity ceased, leaves of this species started to color and gradually fall. In contrast, the stem cambium phenology of *U. pumila* preceded its leaf phenology in the spring, and the onset of cambial activity occurred almost simultaneously in both the trunks and branches. Notably, xylem lignification in the trunks continued even after the leaves began to color and senesce in *U. pumila* (**Figure 5**, **Supplementary Table S2**).

### 3.5 Comparison in xylogenesis between tree trunks and branches

**Figure 6:**
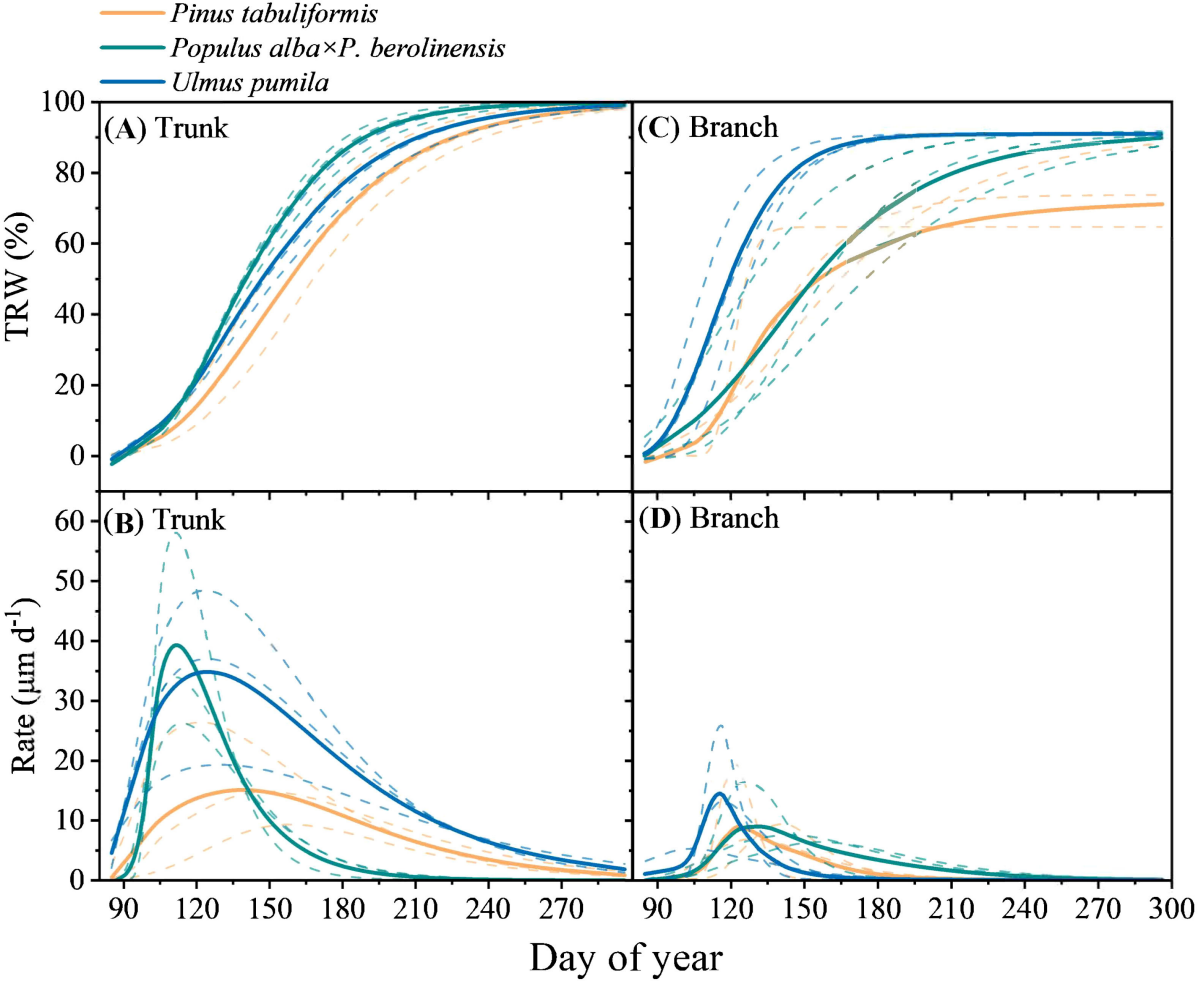
Seasonal pattern of cumulative tree ring width (TRW, %) expressed as **(A, C)** percentage of the total ring width and **(B, D)** growth rates of tree ring width (Rate, μm d-1) of the three studied tree species in the trunk and branches. The curves in **(A, C)** were fitted using Gompertz model and the curves in **(B, D)** show daily growth rates.

The xylogenesis processes in tree trunks and branches exhibited different patterns with the average maximum growth rate of branches occurred earlier than that of the trunks (**Supplementary Figures S2**, **S3**; **Figure 6**). In *P. tabuliformis*, the maximum growth rate of the trunk was 15.4 μm d^-1^ that occurred in mid-May (DOY 138.0), while the maximum growth rate of the branches was 12.5 μm d^-1^ that was recorded in mid-April (DOY 104.0). Notably, the maximum growth rate of the trunk occurred 34 days later than that of the branches. For *P. alba* × *P. berolinensis*, the maximum growth rates were observed at the end of April, with the trunk reaching 40.8 μm d^-1^ on DOY 111.0 and the branches reaching 9.3 μm d^-1^ on DOY 110.0. In *U. pumila*, the maximum growth rate for trunks was 35.7 μm d^-1^ that was observed in early May (DOY 124.0), whereas the maximum growth rate for branches was 17.8 μm d^-1^ that was observed in early April (DOY 100.0). The maximum growth rate of the trunks in *U. pumila* occurred 24 days later than that of the branches.

## 4 Discussion

### 4.1 Adaptive significance of divergence in leaf phenology

The differences in spring leaf phenology between the evergreen conifer species and the deciduous broadleaf species reflect distinct carbon investment-return strategies. Conifer species typically adopt a “slow” strategy, characterized by long lifespan leaves that maintain photosynthesis even as broadleaf species are shedding their leaves (Chen and Xu, 2014; Davis et al., 1999; Givnish, 2002). Although their instantaneous photosynthetic carbon assimilation rate is lower, the ability to retain leaves over the winter allows evergreen conifers to accumulate carbon over a longer growing period (Qi et al., 2021; Zhang et al., 2017, 2013). In early spring, previous year’s needles can be used for photosynthesis under favorable climatic conditions, so there is no selective pressure for early bud burst. In contrast, deciduous tree species generally follow a “quick” strategy, quickly capturing light energy to meet the carbon demands for tree growth within a short growing period (Chen and Xu, 2014; Reich and Cornelissen, 2014), and tend to bud burst earlier. However, early bud burst increases the susceptibility to cold and frost damage (Kollas et al., 2014; Vitasse et al., 2018). For evergreen conifer species, the contribution of the current year needles to photosynthesis is limited, and the benefits of early bud burst are less pronounced. Delaying bud burst helps avoid cold damage and reduces the need for investment in cold resistance for young needles (Hufkens et al., 2012). Thus, the differences in spring leaf phenology between evergreen conifers and deciduous broadleaf tree species represent a balance between maximizing resource acquisition and minimizing costs related to frost resistance (Saxe et al., 2001).

The earlier leaf phenology of the diffuse-porous tree species compared to that of the ring-porous species is likely related to their contrasts in the strategy of coping with the risk of freezing-induced hydraulic dysfunction. Diffuse-porous species exhibit greater resistance to embolism induced by freeze-thaw cycles over the winter, and their xylem maintains relatively high water transport efficiency in early spring (Ameglio et al., 2002; Dai et al., 2020; Hacke and Sauter, 1996). This enables the diffuse-porous species to meet the transpirational demands of leaves after bud burst (Umebayashi et al., 2008), thus facilitating early leaf emergence from the perspective of water transport requirements. In contrast, ring-porous species are highly sensitive to embolism, and their xylem can lose up to 90% or more of stem xylem water transport function over the winter (Davis et al., 1999; Niu et al., 2017; Sperry et al., 1994). Although tree water demand at the stage of bud expansion can depend on the small-sized vessels in the late-woods of previous years that remain functioning over the winter (Savage and Chuine, 2021; Umebayashi et al., 2008; Valdovinos-Ayala et al., 2022), these species primarily rely on the current year early-wood vessels that form in early spring to meet greater hydraulic demands of faster leaf transpiration after leaf expansion (Takahashi et al., 2013). In deed, studies on temperate forest tree species have shown that the rapid expansion of leaves in ring-porous species occurs only after the re-established of an effective water transport system through the formation of new early-wood vessels to support leaf transpiration demands (Yin et al., 2022).

### 4.2 Adaptive significance of divergence in cambium phenology

The differences in the timing of cambial activity among the three studied species seem to be aligned to their differences in sensitivity to xylem embolism induced by freeze-thaw cycles. The early onset of cambial activity in conifer species reflects their strong adaptability to cold temperatures. The xylem of conifers, primarily composed of tracheids, typically has diameters of less than 15 μm and is strongly resistant to embolism induced by freeze-thaw cycles (Rossi et al., 2006c; Sperry and Robson, 2001). This resistance may be crucial for the survival of conifer species in high-altitude and high-latitude environments. The early onset of cambial activity may likely benefit conifers by allowing them to extend the period for xylem growth in such regions with short growing seasons (Charrier et al., 2015; Rossi et al., 2006c). In contrast, the cambial activity of deciduous broadleaf species is delayed relative to that of conifers, which may be attributed to the greater frost sensitivity of vessels compared to tracheids (Davis et al., 1999). Developing vessels with thinner cell walls may be particularly susceptible to embolism induced by freeze-thaw cycles, making early onset of cambial activity less advantageous for broadleaf trees (Granda et al., 2014; Yin et al., 2022). Surprisingly, although the leaves of the ring-porous species emerged later, its cambial activity began significantly earlier than that of the diffuse-porous species, and even earlier than that of the conifer species. This suggests a strong selective pressure favoring early xylem formation in ring-porous species. The large early-wood vessels of ring-porous species, despite very efficient in water transport, are highly sensitive to freeze-thaw cycles and lose much of their water transport function over the winter (Niu et al., 2017; Yin et al., 2018; Zwieniecki et al., 2001). Only when the functional early-wood vessels of the current year are available can the ring-porous trees meet their water transport needs for transpiration (Hacke and Sperry, 2001; Niu et al., 2017). The early onset of cambial activity in ring-porous species, however, increases the risk of embolism induced by freeze-thaw cycles in early spring. As a result, ring-porous species are generally distributed in areas with lower altitudes and latitudes than diffuse-porous species (Yang et al., 2020; Yin et al., 2023).

The differences in chronological sequence of leaf and cambium phenologies in the three tree species are also closely related to their divergence in hydraulic strategy. In conifer trees, cambial activity began about a week earlier than bud burst and they can rely on the perennial needles from the previous years for gas exchange during early spring (Chen et al., 2022; Qian et al., 2024). The early onset of cambial activity can enhance xylem water transport efficiency, which can be critical for effective water transport during the active growing period when transpiration is intense (Pittermann and Sperry, 2006; Sperry and Robson, 2001). In contrast, the bud burst of the diffuse-porous species occurred about 10 days earlier than the onset of cambial activity. This is likely related to the fact that the xylem of diffuse-porous species usually retain a high proportion of its water transport function over the winter (Brodersen and McElrone, 2013). Although the formation of new vessels occurred later than the onset of transpiration, these species can still rely on the vessels from the previous years for water transport (Umebayashi et al., 2008; Yin et al., 2022). The late onset of cambial activity helps avoid the negative effects of early spring frost on the functional xylem, allowing these species to thrive in colder environments compared to ring-porous species (Yang et al., 2020; Yin et al., 2023). In contrast, ring-porous species lose much of the water transport function of their previous early-wood vessels over the winter and must form new early-wood vessels earlier in the spring to restore effective water transport (Copini et al., 2019; Hacke and Sperry, 2001; Niu et al., 2017; Yin et al., 2022). The bud burst closely followed the onset of cambial activity in the ring-porous species, reflecting the double selective pressures tree species of this functional type face, i.e. the need for early cambial activity to meet the transpirational demand for water transport while simultaneously minimizing the risk of early spring frost damage (García-González et al., 2016; Wang et al., 2022).

### 4.3 Divergence in patterns of xylogenesis and the underlying physiology

The differences in radial growth patterns among the three species reflect distinct growth strategies that align with their divergent physiological characteristics. The hydraulic architecture plays a crucial role in regulating leaf transpiration, thereby influencing the photosynthetic capacity and growth rate of the trees (Fan et al., 2012; Ning et al., 2022). Compared to the other two species, the conifer species has a longer growth period and slower instantaneous growth rate. Although conifer species are known for their strong resistance to embolism induced by freeze-thaw cycles (Davis et al., 1999), the relatively lower water transport efficiency of their tracheid-based xylem may have limited their instantaneous growth rate. In contrast, the *Populus* and *Ulmus* species benefit from higher efficiency of water transport in vessel-based xylem, which supports higher photosynthetic carbon assimilation and hence faster growth (Chen et al., 2022; Poorter et al., 2010). Particularly, under conditions of sufficient water supply, the high water transport rate in early-wood vessels of the ring-porous species ensures more effective water delivery to transpiring leaves and hence support high photosynthetic rate (Savage and Chuine, 2021; Wang et al., 2022). The strong coordination between water transport and carbon assimilation ultimately translates into the coordination between hydraulic efficiency and wood growth rate (Poorter et al., 2010; Schuldt et al., 2016). The smaller diameter of the late-wood vessels, along with their higher resistance to embolism and the tree’s longer leaf lifespan, may have played a critical role in enabling *U. pumila* to maintain stem radial growth over a longer period. These patterns illustrate a suite of intercorrelations among different plant functions formed during evolution, including the coordination between water transport and carbon assimilation and the trade-off between hydraulic efficiency and safety.

The patterns of timing of cambial activity in tree trunks and branches of ring-porous and diffuse-porous species reflect the combined effects of both internal and external factors on xylem formation. Ring-porous species are more sensitive to auxin, allowing them to respond strongly to the very low concentrations of auxin released from buds just before bud burst (Aloni et al., 1997). This sensitivity enables the onset of cambial activity to extend rapidly from the upper parts of the stem to the base. Consequently, the first large early-wood vessels appear before bud burst and occur almost simultaneously on the trunk and branches (Kudo et al., 2015; Takahashi et al., 2013; Takahashi and Takahashi, 2016). In contrast, diffuse-porous species are less sensitive to auxin, and their cambiums require higher concentrations of auxin from rapidly growing young leaves to be reactivated, and it usually takes several weeks to extend from the branches to the base of the trunk (Aloni, 2021; Takahashi et al., 2013). However, the timing of cambial activity in diffuse-porous species does not always follow this pattern. Our research shows that in the *Populus* species, the cambial activity began first in the trunk, suggesting that xylem formation may be influenced not only by auxin but also by external environmental factors. For example, Huang et al. (2023) found that a threshold temperature (4.9 ± 1.1°C) drives the cambial activity and xylem formation in Northern Hemisphere conifers. The chronological order of cambial activity in different parts of the tree may also be influenced by tree age, species, and site conditions (Gričar et al., 2017; Li et al., 2013). Therefore, xylem formation and radial growth in trees may only begin when both internal and external conditions are met simultaneously making the patterns more complex.

## 5 Conclusions

Results of the present study indicate a close coordination between leaf and cambium phenologies, which jointly influence the coupling between water and carbon related physiological processes as well as the growth strategies of trees. We identified divergences in chronological sequences of leaf and cambium phenologies among temperate tree species of different wood types. The conifer species exhibited relatively early cambial reactivation and late leaf phenology, which can be associated with its stronger resistance to freezing-induced embolism of its tracheid-based xylem and the evergreen leaf habit. In the diffuse-porous species, leaf phenology preceded the onset of cambial activity, as most of the vessels from the previous year can perform the water transport function in spring, supporting the transpirational water demands of the earlier-emerging leaves, while its relatively late onset of cambial activity effectively avoids the potential adverse effects of early spring frost on the cambium and xylem tissues. In contrast, the earliest onset of cambial activity in the ring-porous species, reflects the high selective pressure to restore its severely hampered stem hydraulic function before leaf flush. The bud burst closely followed the onset of cambial activity in the ring-porous species, indicating that this species faces dual selective pressures, i.e. starting cambial activity early to meet the hydraulic demand for transpiration after leaf flush, while also minimizing the risk of frost damage to cambium and the newly formed xylem tissues in early spring. This study identifies differences in leaf and cambium phenologies among the three temperate tree species with different wood types as well as reveals the underlying physiological mechanisms, particularly from the perspective of xylem hydraulics related to the risk of winter embolism in temperate trees. Given that the number of tree species in each functional group is limited in this study, future research should expand the sample size and incorporate other relevant aspects to confirm the generalizability of the observed patterns. Nevertheless, these findings substantially contribute to a better understanding of the responses of trees to environmental changes and the potential impacts of climate change on forest ecosystems.

## Data Availability

The original contributions presented in the study are included in the article/**Supplementary Material**. Further inquiries can be directed to the corresponding author.

## Supplementary material

Supplementary Table 1 Leaf phenology of the three studied species in 2023. Data are shown as day of year (DOY) ± 1 SE (*n* = 3). Different letters following the numbers indicate significant differences between the three species at *P* < 0.05 level.

Supplementary Table 2 Cambium phenology of the three studied species in 2023. Data are shown as day of year (DOY) ± 1 SE (*n* = 3). Different letters following the numbers indicate significant differences between the trunk and branches within a species at *P* < 0.05 level.

Supplementary Figure 1 Variations in daily temperature, precipitation, relative humidity (RH) and vapour pressure deficit (VPD) in 2023 in Shenyang.

Supplementary Figure 2 The width of **(a)** the cambial zone, **(b)** the enlarging cell zone, **(c)** the wall thickening cell zone, and **(d)** the mature cell zone in tree trunks of the three studied species during 2023. Data are presented as mean ± 1 SE.

Supplementary Figure 3 The width of **(a)** the cambial zone, **(b)** the enlarging cell zone, **(c)** the wall thickening cell zone, and **(d)** the mature cell zone in branches of the three studied species during 2023. Data are presented as mean ± 1 SE.

## References

[B1] Aloni R. (1991). “Wood formation in deciduous hardwood trees,” in Physiology of Trees. Ed. Raghavendra A. S. (Wiley & Sons, New York), 175–197.

[B2] Aloni R. (1992). The control of vascular differentiation. Int. J. Plant Sci. 153, S90–S92. doi: 10.1086/297067

[B3] Aloni R. (2021). “Hormonal control of wood evolution,” in Vascular Differentiation and Plant Hormones (Springer International Publishing, Cham), 273–291p.

[B4] Aloni R. Alexander J. D. Tyree M. T. (1997). Natural and experimentally altered hydraulic architecture of branch junctions in *Acer saccharum* Marsh. and *Quercus velutina* Lam. trees. Trees 11, 255–264. doi: 10.1007/PL00009672

[B5] Aloni R. Peterson C. A. (1997). Auxin promotes dormancy callose removal from the phloem of *Magnolia kobus* and callose accumulation and earlywood vessel differentiation in Quercus robur. J. Plant Res. 110, 37. doi: 10.1007/BF02506841 27520042

[B6] Ameglio T. Bodet C. Lacointe A. Cochard H. (2002). Winter embolism, mechanisms of xylem hydraulic conductivity recovery and springtime growth patterns in walnut and peach trees. Tree Physiol. 22, 1211–1220. doi: 10.1093/treephys/22.17.1211 12464574

[B7] Antonucci S. Rossi S. Deslauriers A. Lombardi F. Marchetti M. Tognetti R. (2015). Synchronisms and correlations of spring phenology between apical and lateral meristems in two boreal conifers. Tree Physiol. 35, 1086–1094. doi: 10.1093/treephys/tpv077 26377874

[B8] Badeck F. W. Bondeau A. Böttcher K. Doktor D. Lucht W. Schaber J.. (2004). Responses of spring phenology to climate change. New Phytol. 162, 295–309. doi: 10.1111/j.1469-8137.2004.01059.x

[B9] Begum S. Kudo K. Matsuoka Y. Nakaba S. Yamagishi Y. Nabeshima E.. (2016). Localized cooling of stems induces latewood formation and cambial dormancy during seasons of active cambium in conifers. Ann. Bot. 117, 465–477. doi: 10.1093/aob/mcv181 26703452 PMC4765539

[B10] Brodersen C. R. McElrone A. J. (2013). Maintenance of xylem network transport capacity: a review of embolism repair in vascular plants. Front. Plant Sci. 4, 108. doi: 10.3389/fpls.2013.00108 23630539 PMC3633935

[B11] Charrier G. Ngao J. Saudreau M. Ameglio T. (2015). Effects of environmental factors and management practices on microclimate, winter physiology, and frost resistance in trees. Front. Plant Sci. 6, 259. doi: 10.3389/fpls.2015.00259 25972877 PMC4411886

[B12] Chen Y. Rademacher T. Fonti P. Eckes-Shephard A. H. LeMoine J. M. Fonti M. V.. (2022). Inter-annual and inter-species tree growth explained by phenology of xylogenesis. New Phytol. 235, 939–952. doi: 10.1111/nph.v235.3 35488501 PMC9325364

[B13] Chen Y. T. Xu Z. Z. (2014). Review on research of leaf economics spectrum. Chin. J. Plant Ecol. 38, 1135–1153. doi: 10.3724/SP.J.1258.2014.00108

[B14] Chuine I. (2010). Why does phenology drive species distribution? Philos. Trans. R Soc. Lond B Biol. Sci. 365, 3149–3160. doi: 10.1098/rstb.2010.0142 20819809 PMC2981946

[B15] Copini P. Vergeldt F. J. Fonti P. Sass-Klaassen U. den Ouden J. Sterck F.. (2019). Magnetic resonance imaging suggests functional role of previous year vessels and fibres in ring-porous sap flow resumption. Tree Physiol. 39, 1009–1018. doi: 10.1093/treephys/tpz019 30896019

[B16] Cuny H. E. Rathgeber C. B. Lebourgeois F. Fortin M. Fournier M. (2012). Life strategies in intra-annual dynamics of wood formation: example of three conifer species in a temperate forest in north-east France. Tree Physiol. 32, 612–625. doi: 10.1093/treephys/tps039 22543476

[B17] Dai Y. Wang L. Wan X. (2020). Frost fatigue and its spring recovery of xylem conduits in ring-porous, diffuse-porous, and coniferous species in situ. Plant Physiol. Biochem. 146, 177–186. doi: 10.1016/j.plaphy.2019.11.014 31756604

[B18] Davis S. D. Sperry J. S. Hacke U. G. (1999). The relationship between xylem conduit diameter and cavitation caused by freezing. Am. J. Bot. 86, 1367–1372. doi: 10.2307/2656919 10523278

[B19] Delpierre N. Vitasse Y. Chuine I. Guillemot J. Bazot S. Rutishauser T.. (2016). Temperate, boreal forest tree phenology: from organ-scale processes to terrestrial ecosystem models. Ann. For Sci. 73, 5–25. doi: 10.1007/s13595-015-0477-6

[B20] Deslauriers A. Huang J. G. Balducci L. Beaulieu M. Rossi S. (2016). The contribution of carbon and water in modulating wood formation in black spruce saplings. Plant Physiol. 170, 2072–2084. doi: 10.1104/pp.15.01525 26850274 PMC4825115

[B21] Du H. Liu J. Li M. H. Buntgen U. Yang Y. Wang L.. (2018). Warming-induced upward migration of the alpine treeline in the Changbai Mountains. Northeast China Glob Change Biol. 24, 1256–1266. doi: 10.1111/gcb.2018.24.issue-3 29080270

[B22] Duursma R. A. (2015). Plantecophys-an R package for analysing and modelling leaf gas exchange data. PLoS One 10, e0143346. doi: 10.1371/journal.pone.0143346 26581080 PMC4651500

[B23] Fan Z. X. Zhang S. B. Hao G. Y. Ferry Slik J. W. Cao K. F. (2012). Hydraulic conductivity traits predict growth rates and adult stature of 40 Asian tropical tree species better than wood density. J. Ecol. 100, 732–741. doi: 10.1111/j.1365-2745.2011.01939.x

[B24] García-González I. Souto-Herrero M. Campelo F. (2016). Ring-porosity and earlywood vessels: a review on extracting environmental information through time. IAWA J. 37, 295–314. doi: 10.1163/22941932-20160135

[B25] Givnish T. J. (2002). Adaptive significance of evergreen vs. deciduous leaves: solving the triple paradox. Silva Fenn 36, 703–743. doi: 10.14214/sf.535

[B26] Granda E. Scoffoni C. Rubio-Casal A. E. Sack L. Valladares F. (2014). Leaf and stem physiological responses to summer and winter extremes of woody species across temperate ecosystems. Oikos 123, 1281–1290. doi: 10.1111/more.2014.123.issue-11

[B27] Gričar J. Lavrič M. Ferlan M. Vodnik D. Eler K. (2017). Intra-annual leaf phenology, radial growth and structure of xylem and phloem in different tree parts of Quercus pubescens. Eur. J. For Res. 136, 625–637. doi: 10.1007/s10342-017-1060-5

[B28] Hacke U. Sauter J. J. (1996). Xylem dysfunction during winter and recovery of hydraulic conductivity in diffuse-porous and ring-porous trees. Oecologia 105, 435–439. doi: 10.1007/bf00330005 28307135

[B29] Hacke U. G. Sperry J. S. (2001). Functional and ecological xylem anatomy. Perspect. Plant Ecol. Evol. Syst. 4, 97–115. doi: 10.1078/1433-8319-00017

[B30] Hao G. Y. Wheeler J. K. Holbrook N. M. Goldstein G. (2013). Investigating xylem embolism formation, refilling and water storage in tree trunks using frequency domain reflectometry. J. Exp. Bot. 64, 2321–2332. doi: 10.1093/jxb/ert090 23585669 PMC3654422

[B31] Huang J. G. Zhang Y. Wang M. Yu X. Deslauriers A. Fonti P.. (2023). A critical thermal transition driving spring phenology of Northern Hemisphere conifers. Glob Chang Biol. 29, 1606–1617. doi: 10.1111/gcb.16543 36451586

[B32] Hufkens K. Friedl M. Sonnentag O. Braswell B. H. Milliman T. Richardson A. D. (2012). Linking near-surface and satellite remote sensing measurements of deciduous broadleaf forest phenology. Remote Sens Environ. 117, 307–321. doi: 10.1016/j.rse.2011.10.006

[B33] Jyske T. Holtta T. (2015). Comparison of phloem and xylem hydraulic architecture in *Picea abies* stems. New Phytol. 205, 102–115. doi: 10.1111/nph.2014.205.issue-1 25124270

[B34] Kollas C. Körner C. Randin C. F. (2014). Spring frost and growing season length co-control the cold range limits of broad-leaved trees. J. Biogeogr 41, 773–783. doi: 10.1111/jbi.2014.41.issue-4

[B35] Kudo K. Yasue K. Hosoo Y. Funada R. (2015). Relationship between formation of earlywood vessels and leaf phenology in two ring-porous hardwoods, *Quercus serrata* and *Robinia pseudoacacia*, in early spring. J. Wood Sci. 61, 455–464. doi: 10.1007/s10086-015-1487-6

[B36] Lavrič M. Eler K. Ferlan M. Vodnik D. Gričar J. (2017). Chronological sequence of leaf phenology, xylem and phloem formation and sap flow of *Quercus pubescens* from Abandoned Karst Grasslands. Front. Plant Sci. 8, 314. doi: 10.3389/fpls.2017.00314 28321232 PMC5337753

[B37] Li X. Liang E. Gričar J. Prislan P. Rossi S. Cufar K. (2013). Age dependence of xylogenesis and its climatic sensitivity in Smith fir on the south-eastern Tibetan Plateau. Tree Physiol. 33, 48–56. doi: 10.1093/treephys/tps113 23185065

[B38] Linkosalo T. Häkkinen R. Terhivuo J. Tuomenvirta H. Hari P. (2009). The time series of flowering and leaf bud burst of boreal trees, (1846–2005) support the direct temperature observations of climatic warming. Agric. For Meteorol 149, 453–461. doi: 10.1016/j.agrformet.2008.09.006

[B39] Michelot A. Simard S. Rathgeber C. Dufrene E. Damesin C. (2012). Comparing the intra-annual wood formation of three European species (*Fagus sylvatica*, *Quercus petraea* and *Pinus sylvestris*) as related to leaf phenology and non-structural carbohydrate dynamics. Tree Physiol. 32, 1033–1045. doi: 10.1093/treephys/tps052 22718524

[B40] Morel H. Mangenet T. Beauchêne J. Ruelle J. Nicolini E. Heuret P.. (2015). Seasonal variations in phenological traits: leaf shedding and cambial activity in *Parkia nitida* Miq. and *Parkia velutina* Benoist (Fabaceae) in tropical rainforest. Trees 29, 973–984. doi: 10.1007/s00468-015-1177-4

[B41] Morin X. Roy J. Sonie L. Chuine I. (2010). Changes in leaf phenology of three European oak species in response to experimental climate change. New Phytol. 186, 900–910. doi: 10.1111/j.1469-8137.2010.03252.x 20406403

[B42] Muffler L. Weigel R. Beil I. Leuschner C. Schmeddes J. Kreyling J. (2024). Winter and spring frost events delay leaf-out, hamper growth and increase mortality in European beech seedlings, with weaker effects of subsequent frosts. Ecol. Evol. 14, e70028. doi: 10.1002/ece3.70028 39041017 PMC11260882

[B43] Ning Q. R. Gong X. W. Li M. Y. Hao G. Y. (2022). Differences in growth pattern and response to climate warming between *Larix olgensis* and *Pinus koraiensis* in Northeast China are related to their distinctions in xylem hydraulics. Agric. For Meteorol 312, 108724. doi: 10.1016/j.agrformet.2021.108724

[B44] Niu C. Y. Meinzer F. C. Hao G. Y. (2017). Divergence in strategies for coping with winter embolism among co-occurring temperate tree species: the role of positive xylem pressure, wood type and tree stature. Funct. Ecol. 31, 1550–1560. doi: 10.1111/fec.2017.31.issue-8

[B45] Nord E. A. Lynch J. P. (2009). Plant phenology: a critical controller of soil resource acquisition. J. Exp. Bot. 60, 1927–1937. doi: 10.1093/jxb/erp018 19286918

[B46] O’Reilly C. Owens J. N. (1989). Shoot, needle, and cambial growth phenology and branch tracheid dimensions in provenances of lodgepole pine. Can. J. For Res. 19, 599–605. doi: 10.1139/x89-094

[B47] Perrin M. Rossi S. Isabel N. (2017). Synchronisms between bud and cambium phenology in black spruce: early-flushing provenances exhibit early xylem formation. Tree Physiol. 37, 593–603. doi: 10.1093/treephys/tpx019 28338976

[B48] Pittermann J. Sperry J. S. (2006). Analysis of freeze-thaw embolism in conifers. The interaction between cavitation pressure and tracheid size. Plant Physiol. 140, 374–382. doi: 10.1104/pp.105.067900 16377751 PMC1326058

[B49] Pittermann J. Sperry J. S. Hacke U. G. Wheeler J. K. Sikkema E. H. (2005). Torus-margo pits help conifers compete with angiosperms. Science 310, 1924–1924. doi: 10.1126/science.1120479 16373568

[B50] Poorter L. McDonald I. Alarcon A. Fichtler E. Licona J. C. Pena-Claros M.. (2010). The importance of wood traits and hydraulic conductance for the performance and life history strategies of 42 rainforest tree species. New Phytol. 185, 481–492. doi: 10.1111/j.1469-8137.2009.03092.x 19925555

[B51] Puchałka R. Koprowski M. Gričar J. Przybylak R. (2017). Does tree-ring formation follow leaf phenology in Pedunculate oak (*Quercus robur* L.)? Eur. J. For Res. 136, 259–268. doi: 10.1007/s10342-017-1026-7

[B52] Qi J. H. Fan Z. X. Fu P. L. Zhang Y. J. Sterck F. (2021). Differential determinants of growth rates in subtropical evergreen and deciduous juvenile trees: carbon gain, hydraulics and nutrient-use efficiencies. Tree Physiol. 41, 12–23. doi: 10.1093/treephys/tpaa131 33080622

[B53] Qian N. Gao H. Xu Z. Song C. Dong C. Zeng W.. (2023). Cambial phenology and wood formation of Korean pine in response to climate change in Changbai Mountain, Northeast China. Dendrochronologia 77, 126045. doi: 10.1016/j.dendro.2022.126045

[B54] Qian N. Xu Z. Gao H. Song C. Dong C. Hu B.. (2024). Linkages between intra-annual radial growth and photosynthetic production of four main species in a temperate forest in Northeast China. Agric. For Meteorol 345, 109866. doi: 10.1016/j.agrformet.2023.109866

[B55] Reich P. B. Cornelissen H. (2014). The world-wide ‘fast–slow’ plant economics spectrum: a traits manifesto. J. Ecol. 102, 275–301. doi: 10.1111/jec.2014.102.issue-2

[B56] Rossi S. Anfodillo T. Cufar K. Cuny H. E. Deslauriers A. Fonti P.. (2016). Pattern of xylem phenology in conifers of cold ecosystems at the Northern Hemisphere. Glob Chang Biol. 22, 3804–3813. doi: 10.1111/gcb.2016.22.issue-11 27082838

[B57] Rossi S. Anfodillo T. Menardi R. (2006a). Trephor: a new tool for sampling microcores from tree stems. IAWA J. 27, 89–97. doi: 10.1163/22941932-90000139

[B58] Rossi S. Deslauriers A. Anfodillo T. (2006b). Assessment of cambial activity and xylogenesis by microsampling tree species: an example at the Alpine timberline. IAWA J. 27, 383–394. doi: 10.1163/22941932-90000161

[B59] Rossi S. Deslauriers A. Anfodillo T. Morin H. Saracino A. Motta R.. (2006c). Conifers in cold environments synchronize maximum growth rate of tree-ring formation with day length. New Phytol. 170, 301–310. doi: 10.1111/j.1469-8137.2006.01660.x 16608455

[B60] Rossi S. Morin H. Deslauriers A. (2012). Causes and correlations in cambium phenology: towards an integrated framework of xylogenesis. J. Exp. Bot. 63, 2117–2126. doi: 10.1093/jxb/err423 22174441 PMC3295399

[B61] Savage J. A. Chuine I. (2021). Coordination of spring vascular and organ phenology in deciduous angiosperms growing in seasonally cold climates. New Phytol. 230, 1700–1715. doi: 10.1111/nph.v230.5 33608961

[B62] Saxe H. Cannell M. G. R. Johnsen O. Ryan M. G. Vourlitis G. (2001). Tree and forest functioning in response to global warming. New Phytol. 149, 369–399. doi: 10.1046/j.1469-8137.2001.00057.x 33873342

[B63] Schuldt B. Knutzen F. Delzon S. Jansen S. Muller-Haubold H. Burlett R.. (2016). How adaptable is the hydraulic system of European beech in the face of climate change-related precipitation reduction? New Phytol. 210, 443–458. doi: 10.1111/nph.13798 26720626

[B64] Song J. Trueba S. Yin X. H. Cao K. F. Brodribb T. J. Hao G. Y. (2022). Hydraulic vulnerability segmentation in compound-leaved trees: Evidence from an embolism visualization technique. Plant Physiol. 189, 204–214. doi: 10.1093/plphys/kiac034 35099552 PMC9070814

[B65] Sperry J. S. Nichols K. L. Sullivan J. E. Eastlack S. E. (1994). Xylem embolism in ring-porous, diffuse-porous, and coniferous trees of northern Utah and interior Alaska. Ecology 75, 1736–1752. doi: 10.2307/1939633

[B66] Sperry J. S. Robson D. J. (2001). Xylem cavitation and freezing in conifers. In: Bigras FJ Colombo SJ (eds) Conifer Cold Hardiness. Tree Physiol. vol 1. Springer,, Dordrecht. doi: 10.1007/978-94-015-9650-3_5

[B67] Sperry J. S. Sullivan J. E. (1992). Xylem embolism in response to freeze-thaw cycles and water stress in ring-porous, diffuse-porous, and conifer species. Plant Physiol. 100, 605–613. doi: 10.1104/pp.100.2.605 16653035 PMC1075601

[B68] Steppe K. Sterck F. Deslauriers A. (2015). Diel growth dynamics in tree stems: linking anatomy and ecophysiology. Trends Plant Sci. 20, 335–343. doi: 10.1016/j.tplants.2015.03.015 25911419

[B69] Takahashi S. Okada N. Nobuchi T. (2013). Relationship between the timing of vessel formation and leaf phenology in ten ring-porous and diffuse-porous deciduous tree species. Ecol. Res. 28, 615–624. doi: 10.1007/s11284-013-1053-x

[B70] Takahashi S. Takahashi E. (2016). Timing of vessel formation in twigs and trunks in relation to porosity and leaf flushing. IAWA J. 37, 16–27. doi: 10.1163/22941932-20160118

[B71] Tyree M. T. Ewers F. W. (1991). The hydraulic architecture of trees and other woody plants. New Phytol. 119, 345–360. doi: 10.1111/j.1469-8137.1991.tb00035.x

[B72] Umebayashi T. Utsumi Y. Koga S. Inoue S. Fujikawa S. Arakawa K.. (2008). Conducting pathways in north temperate deciduous broadleaved trees. IAWA J. 29, 247–263. doi: 10.1163/22941932-90000184

[B73] Valdovinos-Ayala J. Robles C. Fickle J. C. Pérez-de-Lis G. Pratt R. B. Jacobsen A. L. (2022). Seasonal patterns of increases in stem girth, vessel development, and hydraulic function in deciduous tree species. Ann. Bot. 130, 355–365. doi: 10.1093/aob/mcac032 35274669 PMC9486900

[B74] Vitasse Y. Schneider L. Rixen C. Christen D. Rebetez M. (2018). Increase in the risk of exposure of forest and fruit trees to spring frosts at higher elevations in Switzerland over the last four decades. Agric. For Meteorol 248, 60–69. doi: 10.1016/j.agrformet.2017.09.005

[B75] Wang A. Y. Cui H. X. Gong X. W. Guo J. J. Wu N. Hao G. Y. (2022). Contrast in vulnerability to freezing-induced xylem embolism contributes to divergence in spring phenology between diffuse- and ring-porous temperate trees. For Ecosyst. 9, 100070. doi: 10.1016/j.fecs.2022.100070

[B76] Yang D. Wang A. Y. Zhang J. L. Bradshaw C. J. A. Hao G. Y. (2020). Variation in stem xylem traits is related to differentiation of upper limits of tree species along an elevational gradient. Forests 11, 349. doi: 10.3390/f11030349

[B77] Yang D. Zhang Y. J. Song J. Niu C. Y. Hao G. Y. (2019). Compound leaves are associated with high hydraulic conductance and photosynthetic capacity: evidence from trees in Northeast China. Tree Physiol. 39, 729–739. doi: 10.1093/treephys/tpy147 30668831

[B78] Yin X. H. Hao G. Y. Sterck F. (2022). A trade-off between growth and hydraulic resilience against freezing leads to divergent adaptations among temperate tree species. Funct. Ecol. 36, 739–750. doi: 10.1111/1365-2435.13991

[B79] Yin X. H. Hao G. Y. Sterck F. (2023). Ring- and diffuse-porous tree species from a cold temperate forest diverge in stem hydraulic traits, leaf photosynthetic traits, growth rate and altitudinal distribution. Tree Physiol. 43, 722–736. doi: 10.1093/treephys/tpad008 36715627

[B80] Yin X. H. Sterck F. Hao G. Y. (2018). Divergent hydraulic strategies to cope with freezing in co-occurring temperate tree species with special reference to root and stem pressure generation. New Phytol. 219, 530–541. doi: 10.1111/nph.2018.219.issue-2 29682759

[B81] Zhang J. Gou X. Pederson N. Zhang F. Niu H. Zhao S.. (2018). Cambial phenology in *Juniperus przewalskii* along different altitudinal gradients in a cold and arid region. Tree Physiol. 38, 840–852. doi: 10.1093/treephys/tpx160 29401316

[B82] Zhang Y. J. Meinzer F. C. Qi J. H. Goldstein G. Cao K. F. (2013). Midday stomatal conductance is more related to stem rather than leaf water status in subtropical deciduous and evergreen broadleaf trees. Plant Cell Environ. 36, 149–158. doi: 10.1111/j.1365-3040.2012.02563.x 22715809

[B83] Zhang Y. J. Sack L. Cao K. F. Wei X. M. Li N. (2017). Speed versus endurance trade-off in plants: leaves with higher photosynthetic rates show stronger seasonal declines. Sci. Rep. 7, 42085. doi: 10.1038/srep42085 28186201 PMC5301202

[B84] Zwieniecki M. A. Melcher P. J. Michele Holbrook N. (2001). Hydrogel control of xylem hydraulic resistance in plants. Science 291, 1059–1062. doi: 10.1126/science.1057175 11161220

