# Supplementary material for: Divergence in leaf and cambium phenologies among three temperate tree species of different wood types with special reference to xylem hydraulics
Source: Front Plant Sci. 2025 Mar 3;16:1562873. doi: 10.3389/fpls.2025.1562873 (PMC11911365; doi:10.3389/fpls.2025.1562873)
Supplement: Supplementary Table 1 — Leaf phenology of the three studied species in 2023. Data are shown as day of year (DOY) ± 1 SE (n = 3). Different letters following the numbers indicate significant differences between the three species at P < 0.05 level. [file Supplementaryfile1.docx]

**Supplemental Information for:**

**Divergence in leaf and cambium phenologies among three temperate tree species of different wood types with special reference to xylem hydraulics**

Ai-Ying Wang^1,2,†^, Si-Qi Li^1,2,†^, Han-Xiao Cui^3,4^, Ya-Nan Liu^3,4^, Yi-Jun Lu^1,2^, Guang-You Hao^3,*^

^1^College of Life Science and Bioengineering, Shenyang University, Shenyang, Liaoning 110044, China;

^2^Liaoning Key Laboratory of Urban Integrated Pest Management and Ecological Security, College of Life Science and Bioengineering, Shenyang University, Shenyang 110044, Liaoning, China;

^3^CAS Key Laboratory of Forest Ecology and Silviculture, Institute of Applied Ecology, Chinese Academy of Sciences, Shenyang 110016, China;

^4^College of Resources and Environment, University of Chinese Academy of Sciences, Beijing 100049, China.

^†^These authors contributed equally to the manuscript.

^*^Author for correspondence

Guang-You Hao

Tel: +86 24 8397 0374

E-mail address: [haogy@iae.ac.cn](mailto:haogy@iae.ac.cn)

Table S1 Leaf phenology of the three studied species in 2023.

| Leaf phenology | | *Pinus tabuliformis* | *Populus alba* × *Populus berolinensis* | *Ulmus pumila* |
| --- | --- | --- | --- | --- |
| Spring | Swollen buds | 98.0±0.0 a | 87.3±1.3 c | 92.3±0.3 b |
|  | Bud break | 113.7±0.7 a | 92.0±0.6 b | 93.3±0.9 b |
|  | Leaf emergence | 123.7±1.8 a | 93.3±0.3 b | 95.3±0.3 b |
|  | Leaf development | 134.0±0.0 a | 94.3±0.3 b | 97.0±0.0 b |
|  | Full leaf unfolding | 143.7±2.7 a | 96.0±1.5 b | 98.0±0.6 b |
| Autumn | Initiation of autumn colouring | —— | 247.0±0.0 b | 285.7±6.7 a |
|  | Leaf fall | —— | 291.3±4.7 a | 311.0±0.0 a |

Data are shown as day of year (DOY) ± 1 SE (*n* = 3). Different letters following the numbers indicate significant differences between the three species at *P* < 0.05 level .

Table S2 Cambium phenology of the three studied species in 2023.

| Cambial phenology | *Pinus tabuliformis* | | *Populus alba* × *Populus berolinensis* | | *Ulmus pumila* | |
| --- | --- | --- | --- | --- | --- | --- |
|  | trunk | branch | trunk | branch | trunk | branch |
| first enlarging cell | 92.0±0.0 a | 92.0±0.0 a | 103.3±5.9 a | 112.7±4.1 a | 89.7±2.3 a | 88.5±3.5 a |
| first wall-thickening cell | 133.7±11.0 a | 122.0±6.4 a | 126.7±4.1 a | 128.7±2.7 a | 106.0±0.0 a | 105.3±4.1 a |
| first mature cell | 143.3±4.7 a | 131.3±8.5 a | 136.3±2.3 a | 131.3±2.7 a | 120.0±0.0 a | 122.0±2.0 a |
| last enlarging cell | 284.7±11.3 a | 252.7±9.3 a | 192.0±16.2 a | 196.7±9.3 a | 262.0±0.0 a | 168.7±4.7 b |
| last wall-thickening cell | 284.7±11.3 a | 262.0±0.0 a | 201.3±9.3 a | 206.0±0.0 a | 273.3±11.3 a | 168.7±4.7 b |

Data are shown as day of year (DOY) ± 1 SE (*n* = 3). Different letters following the numbers indicate significant differences between the trunk and branches within a species at *P* < 0.05 level .


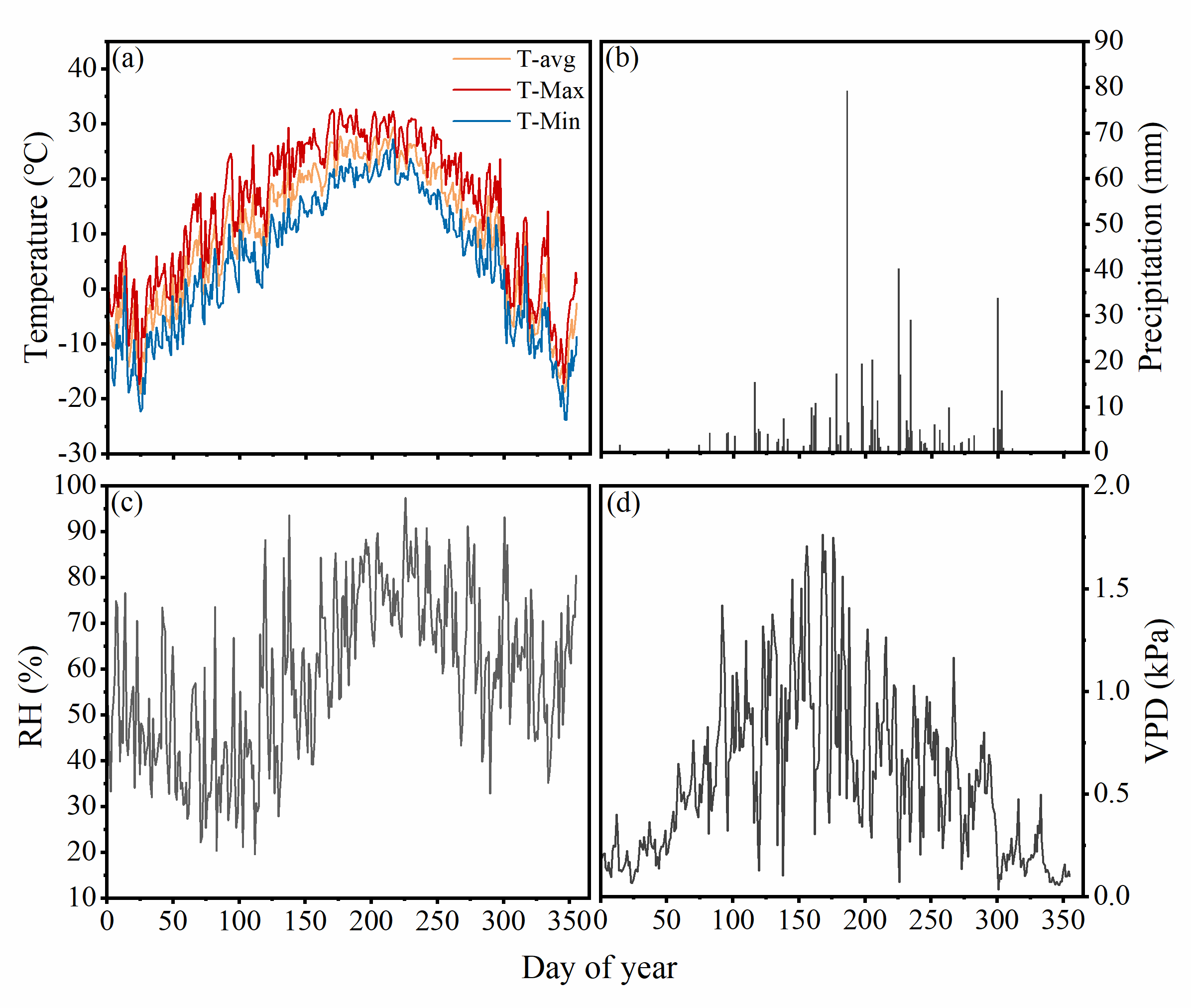


**Fig. S1** Variations in daily temperature, precipitation, relative humidity (RH) and vapour pressure deficit (VPD) in 2023 in Shenyang.


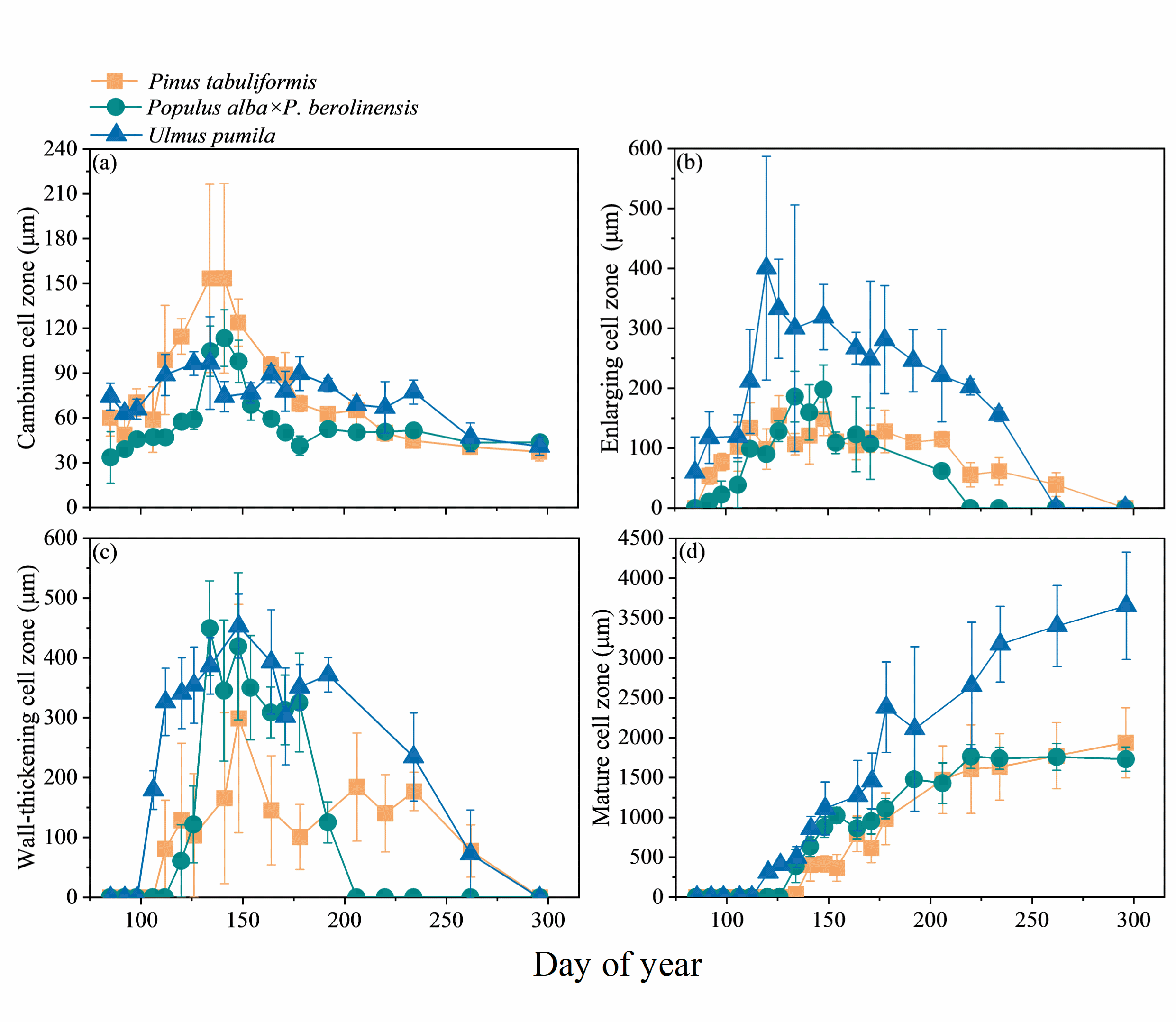


**Fig. S2** The width of (a) the cambial zone, (b) the enlarging cell zone, (c) the wall thickening cell zone, and (d) the mature cell zone in tree trunks of the three studied species during 2023. Data are presented as mean ± 1 SE.


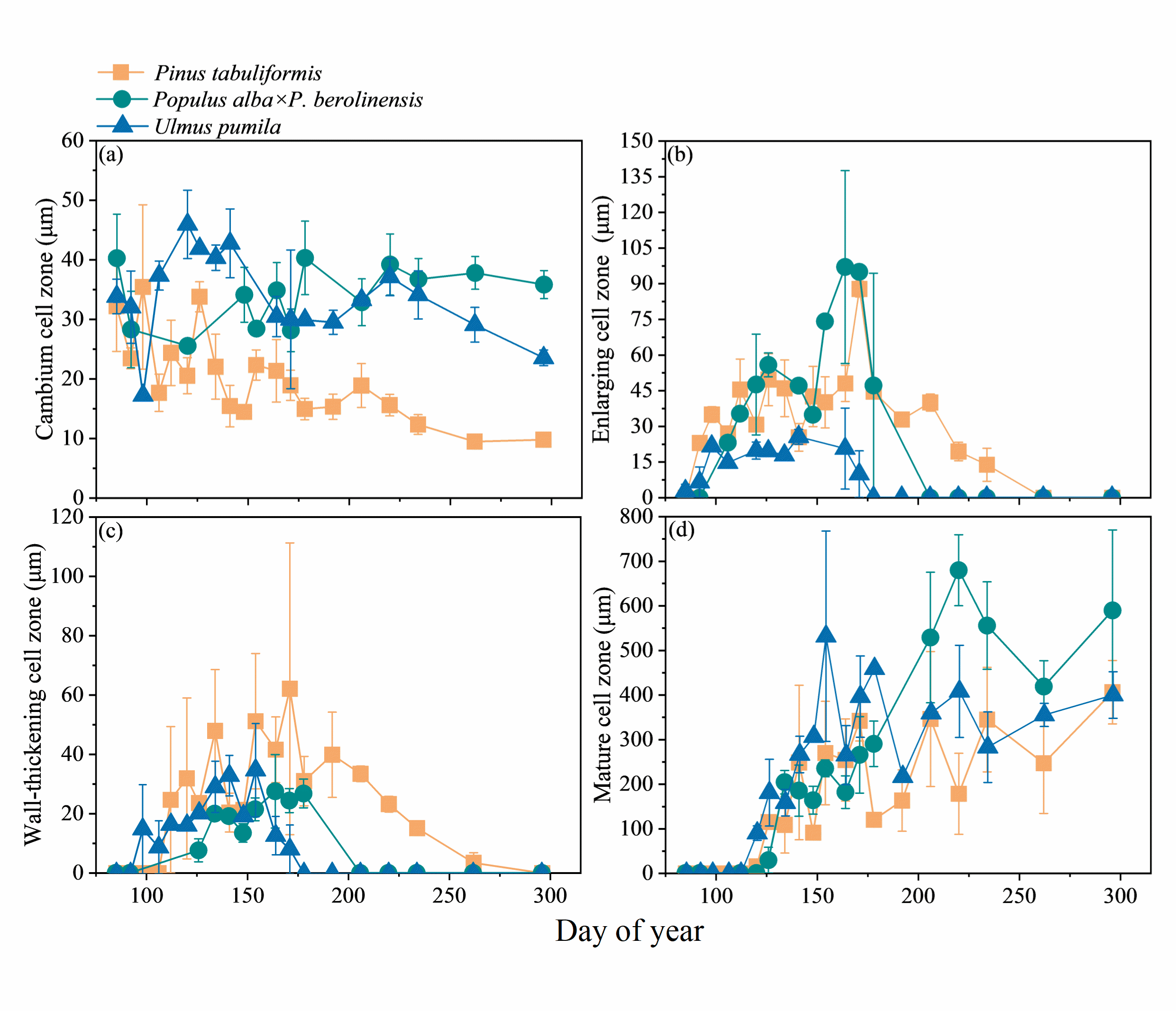


**Fig. S3** The width of (a) the cambial zone, (b) the enlarging cell zone, (c) the wall thickening cell zone, and (d) the mature cell zone in branches of the three studied species during 2023. Data are presented as mean ± 1 SE.
